# Is the bonding of self-adhesive cement sensitive to root region and curing mode?

**DOI:** 10.1590/1678-77572015-0430

**Published:** 2017

**Authors:** Thaynara Faelly BOING, Giovana Mongruel GOMES, João Carlos GOMES, Alessandra REIS, Osnara Maria Mongruel GOMES

**Affiliations:** 1 Universidade Estadual de Ponta Grossa, Departamento de Odontologia, Ponta Grossa, PR, Brazil.

**Keywords:** Resin cements, Raman spectroscopy, Dental bonding

## Abstract

**Objectives:**

To evaluate the influence of two curing techniques on the degree of conversion (DC) of resin cements and on bond strength (BS) of fiber posts in different regions of root dentin.

**Material and Methods:**

Twenty single-rooted premolars were endodontically treated, and the post spaces were prepared. The roots were randomly divided into two groups (n=10), according to the activation mode of the resin cement RelyX™ U200 (3M ESPE Saint Paul, MN, USA): conventional (continuous activation mode) and soft-start activation mode (Ramp). The posts (WhitePost DC/FGM) were cemented according to the manufacturer’s recommendations and, after one week, the roots were cross-sectioned into six discs each of 1-mm thickness, and the cervical, medium, and apical thirds of the root canals were identified. The DC was evaluated under micro-Raman spectroscopy and the BS was evaluated by the push-out test. The data were analyzed by two-way ANOVA and Tukey’s test (α=0.05).

**Results:**

Neither the activation mode nor the root regions affected the DC of the resin cement. Higher BS was achieved in the soft-start group (p=0.036); lower BS was observed in the apical third compared to the other root regions (p<0.001). Irrespective of the activation mode and root region, the mixed failure mode was the most prevalent.

**Conclusion:**

The BS of fiber posts to root canals can be improved by soft-started polymerization. The DC was not affected by the curing mode.

## Introduction

Endodontically treated teeth usually demonstrate an extensive loss of dental structure and require the use of intraradicular retainers and filling cores to hold the final restoration^29^. In this context, the cementation of fiber posts in endodontically treated canals results in restorations that resemble the natural dental structure because the modulus of elasticity of the adhesive materials and fiber posts are similar to that of dentin^14^.

Unfortunately, several factors can affect the adhesion of fiber posts to root dentin, including the histological and anatomical characteristics of the root canal, density and orientation of the dentinal tubules in the different root canal regions^11^, as well as accessibility to the different root canal regions^12^. Different areas of the same root canal also do not respond to acid etching, and thus the ability of adhesion to root dentin may be different at different depths in the same root canal^11^. Higher bond strength values at the cervical third are generally expected due to the ease of conditioning, and polymerization of the cements in this region^9^. However, this is still controversial^3^.

Failures in the luting process of fiber posts are still the major clinical failure^18^. The polymerization shrinkage of these materials may exceed their bond strength, resulting in gaps forming at the dentin-resin cement interface, loss of retention and the displacement of the posts^4^. This may be caused by the stress that results from the polymerization shrinkage generated in the root canal by the composition of resin cements and different light curing techniques^10,23^.

During the pre-gel phase, the shrinkage stress is low due to the high flowability of the resin material. However, when the gel point is reached, the material’s flowability is lost; then the stress generated is transferred to the remaining tooth structure, causing adhesive failures and several other adverse consequences such as tooth fracture and microcracks in the material itself^6,23^.

This situation is even worse due to the high and unfavorable cavity configuration factor (C-factor) of the root space^28^. Some authors have attributed the gap formation and low bond strength to the high C-factor of the root space^26^. One way to control this polymerization shrinkage stress is by reducing the light intensity of the curing unit during the polymerization of the material. The use of soft-start activation provides low light intensity during the initial seconds of activation, increasing the period during which the resinous material remains in the pre-gel phase. Delaying photoactivation decreased the studied post-gel shrinkage^23^ and polymerization shrinkage stress^8^.

Although this technique has shown promising results when employed in composite resin specimens^17^, to the extent of the author’s knowledge, no study has yet evaluated this technique for fiber post cementation. Thus, this *in vitro* study, aimed to compare the degree of conversion and the bond strength of fiber posts in different regions of root dentin, using both conventional and soft-start polymerization techniques. The null hypothesis tested was that the degree of conversion and bond strength of the resin cement is not affected by curing mode or by root region.

## Material and methods

The research project was approved by the Ethics Committee of the Dental School of the State University of Ponta Grossa, under protocol number 109.876. Twenty extracted human mandibular premolars were stored in distilled water at 4°C and used within 6 months from the extraction time. The inclusion criteria was that teeth were absent of restoration, caries or root cracks, absent of previous endodontic treatments, posts or crowns and absent of severe root curvatures. Further, a root length of 14±1 mm measured from the cement-enamel junction was required.

### Specimen preparation

Teeth were transversally sectioned at the cement-enamel junction using a low-speed diamond saw (Isomet 1000, Buehler, Lake Bluff, IL, USA). Endodontic access was made using a tapered fissure bur with a high-speed handpiece and water spray. The working length was established by inserting a #10 Flexofile (Maillefer, Dentsply Ind. e Com. Ltda., Petrópolis, RJ, Brazil) into each canal until it was visible at the apical foramen. One millimeter was subtracted from this length to establish the working length. A crown-down technique was used for instrumentation with Gates Glidden drills #2 to #4 (Maillefer, Dentsply Ind. e Com. Ltda., Petrópolis, RJ, Brazil). Apical enlargement was performed to size 40, 0.2 taper (Maillefer, Dentsply Ind. e Com. Ltda., Petrópolis, RJ, Brazil). Irrigation was performed after every change of instrument by alternating solutions of 5 ml of 1% NaOCl and 5 ml of 17% EDTA (Biodinâmica Química e Farmacêuitca Ltda., Ibiporã, PR, Brazil) for 5 minutes. Roots were dried with paper points (Maillefer, Dentsply Ind. e Com. Ltda., Petrópolis, RJ, Brazil) and filled with a resin-based sealer (AH Plus, Dentsply DeTrey, Konstanz, Germany) and gutta-percha points using the warm vertical condensation technique. The root access was provisionally filled with conventional glass-ionomer cement (Vitro Fil LC, Nova DFL, Taquara, RJ, Brazil). The roots were stored at 37°C and 100% humidity for one week.

After one week, the gutta-percha was removed using the Gates Glidden burs (Maillefer, Dentsply Ind. e Com. Ltda., Petrópolis, RJ, Brazil), leaving 4 mm of the apical seal and the post space was prepared with a low-speed bur provided by the post manufacturer (WhitePost DC #1, FGM, Joinvile, SC, Brazil) to a fixed depth of 10 mm from the cement-enamel junction. The root canals were irrigated with 10 mL of distilled water and dried with paper points (Maillefer, Dentsply Ind. e Com. Ltda., Petrópolis, RJ, Brazil).

### Experimental groups

At this point, the teeth were randomly divided into 2 groups (n=10) according to the activation mode of the resin cement in the root canal. In half of the teeth, a conventional activation mode (continuous light intensity, energy density of 40 J/s) was employed, while in the other half a soft-start polymerization (with initial low light intensity and an energy density of approximately 38.8 J/s) was employed. The light intensity of the device was measured before the beginning of the experiment using a Led Kondortech radiometer (Kondortech Equip. Odontológicos. Ltda – São Carlos, SP, Brazil). Each glass-fiber post was horizontally sectioned with a water-cooled diamond rotary cutting instrument (#2200 diamond bur, KG Sorensen, Barueri, SP, Brazil) so that a total length of 13 mm remained. The fiber posts were cleaned with 70% alcohol for 5 s. Ten millimeters of the post lengths were cemented inside the root canal, while the remaining cervical 3 mm served as a guide to standardize the distance of the light-curing device from the cervical root region.

All the fiber posts (WhitePost DC #1, FGM) were cemented with a dual, self-adhesive resin cement RelyX™ U200 (3M ESPE, Saint Paul, MN, USA), which was mixed according to the manufacturer’s instructions and introduced into the root canal space with a Centrix syringe (DFL, Rio de Janeiro, RJ, Brazil) before seating the fiber post. After the fiber post was seated, the excess resin cement was removed.

Then, the resin cement was immediately polymerized. In the conventional activation mode, a light intensity of 1200 mW/cm^2^ remained constant throughout the exposure time of 40 s. In the soft-start group, the light intensity increased linearly from 0 to 1200 mW/cm^2^ in the first 5 s of exposure time and remained at 1200 mW/cm^2^ in the next 35 s. The light curing unit LED Raddi Plus (SDI Limited, Victoria, Australia) was employed in this experiment.

After the post luting procedures, the roots with cemented posts were covered with the conventional glass-ionomer cement, (Vitro Fil LC, Nova DFL, Taquara, RJ, Brazil), and all samples subsequently were stored in water at 37°C for one week.

### Sample preparation for Raman spectroscopy and push-out tests

The roots were placed in separate polyvinylchloride tubes and embedded in a Duralay acrylic resin (Reliance Dental, Alsip, IL, USA). The portion of each root that contained the bonded fiber post was sectioned perpendicular to the long axis into six 1-mm-thick slices. An Isomet 1000 precision cutter (Buehler, Lake Bluff, IL, USA) was used under water cooling to create two cervical, two medium, and two apical slices of each tooth. Then, all specimens were observed with a light stereomicroscope at 10X magnification in order to identify any artifacts caused by the sectioning procedure. If any defects were observed, the slices were discarded.

### Evaluation of the degree of conversion

One slice of each third of all teeth was submitted to a micro-Raman spectrometer (Senterra, BrukerOptik GmbH, Ettlingen, Germany) evaluation. After polishing and cleaning, each slice was placed under the microscope of the spectrometer. The micro-Raman spectrometer was first calibrated to zero and then to the coefficient values using a silicon sample. The following micro-Raman parameters were used: 20 mW neon laser with a 532 nm wavelength, spatial resolution of 3 µm, spectral resolution of 5 cm^-1^, accumulation time of 30 s with 6 co-additions, and 100x magnification (Olympus UK, London, UK) to 1-µm beam diameter. In each slice, the spectra were taken from 3 random areas, and a mean of the measures was used to represent the degree of conversion *per* slice.

The Raman spectra at 1,637 cm^-1^ indicates unreacted C=C double bonds of the adhesive, while at 1,608 cm^-1^ it represents the C-C bonds in aromatic rings in the Bis-GMA molecules, used as internal reference peaks. The spectra of the uncured resin cement were also recorded. For this purpose the uncured resin cement was placed on a glass slide and taken for the Raman spectroscopy. The ratios of the area at 1,637 and 1,608 cm^-1^ for both the uncured and cured resin cement allowed for the calculation of the degree of conversion of the material according to the following equations:

(1) Rcured = Peak area 1,637 cm^-1^/ Peak area 1,608 cm^-1^

(2) Runcured = Peak area 1,637 cm^-1^/ Peak area 1,608 cm^-1^

(3) Degree of conversion (%) = (1 – Rcured/Runcured) x 100

### Evaluation of bond strength

All slices, including the one that was used in the micro-Raman analysis, were submitted to a push-out test. The thickness of each slice was measured with a Mitutoyo digital caliper (Kyoto, Japan) with an accuracy of 0.01 mm. The slices were also photographed on both sides with an optical microscope (Olympus, model BX 51, Tokyo, Japan) at 40X magnification in order to calculate the cervical and apical diameters of the root canal (post + resin cement)^14^ for the calculation of the individual bonding areas. This measurement was taken with the UTHSCSA ImageTool 3.0 software (University of Texas Health Science Center, San Antonio, TX, USA).

Each specimen (slice) was subjected to a push-out bond strength test using a universal testing machine (AG-I, Shimadzu Autograph, Tokyo, Japan) at a crosshead speed of 0.5 mm/min. The load was applied in the apical-cervical direction until post dislodgement. Care was taken to center the push-out pin on the center of the post surface without stressing the surrounding post space walls. Different sizes of punch pins were used to match the diameter of the post at the different root thirds. Three sizes of punch pins were selected, one representative for each root canal region: cervical (1.4 mm), medium (1.0 mm) and apical (0.6 mm).

The maximum failure load was recorded in Newtons and converted into MPa by dividing the applied load by the bonded area (lateral area of the root canal). The bonded area was the lateral surface of a truncated cone, and was calculated using the formula: LS = π(R + r)[(h2 + (R – r)2]^1^, where π= 3.14, R= cervical root canal radius (cervical post + resin cement radius), r= apical root canal radius (apical post + resin cement radius), and h= root slice thickness^13^.

### Failure mode analysis

After the push-out evaluation, the failure modes of all specimens were evaluated under a stereomicroscope (40X magnification), only to identify the main substrates where the failures occurred (dentin, resin cement, fiber post or mixed). Two independent and calibrated operators analyzed each fractured specimen. If any disagreement occurred between the evaluators, a consensus had to be obtained by discussion.

Then, according to the failure mode the samples were classified as either: 1. adhesive failure between dentin and resin cement; 2. adhesive failure between resin cement and post; 3. cohesive failure within resin cement; 4. cohesive failure within the post; 5. cohesive failure within dentin; 6. mixed failure^4,5,13,29^.

### Statistical analysis

Before running the parametric statistical analysis, we tested whether or not the assumptions of normality of the data and equality of variances were valid, using the Shapiro-Wilk and Barlett’s tests at an alpha of 5%.

The data obtained on the degree of conversion and bond strength were subjected to two-way repeated measures ANOVA and Tukey’s test at a significance level of 5%. The repeated factor was the root third. The fixed factors were the activation mode (continuous or soft-start) and root region (cervical, medium and apical). The data of the failure modes were compared through a chi-squared test (α=0.05). All calculations were performed using the SPSS^®^ statistical software (Statistical Package for the Social Sciences, version 21.0 Mac, SPSS Inc., Chicago, IL, USA).

## Results

None of the specimens observed presented artifacts caused by the sectioning procedure, therefore all slices were tested.

### Degree of conversion

Neither the main factors [activation mode (p=0.362) and root region (p=0.291)] nor the cross-product interaction (p=0.949) influenced the degree of conversion of the resin cement. The degree of conversion of all groups was statistically similar (Table 1).

**Table 1:** Mean and standard deviation of the degree of conversion (%) of the resin cement using the different activation modes (continuous and soft-start) for the different root regions (cervical, medium and apical)

Activation mode	Root region		
	Cervical	Medium	Apical
Continuous	72.4±6.9	77.0±7.8	73.6±7.0
Soft-Start	74.1±8.9	78.2±5.5	74.7±7.7

### Bond strength

The average values of bond strength in MPa (mean and standard deviation) can be seen in Table 2. The cross-product interaction was not significant (p=0.634), but the main factors activation mode (p=0.036) and root third (p<0.001) were. Higher bond strength values were obtained for the soft-start activation mode and in the apical third of the root canal.

**Table 2:** Mean and standard deviation of bond strength (MPa) of fiber posts to root dentin using the different activation modes (continuous and soft-start) for the different root regions (cervical, medium and apical)

Activation mode	Root region		
	Cervical	Medium	Apical
Continuous	16.2±4.1^B^	17.2±4.4^B^	25.8±7.4^A^
Soft-Start	20.2±4.2^B^	22.1±7.4^B^	26.9±6.3^A^

Means identified with the same uppercase letters are not statistically different

### Failure mode analysis

**Figure 1:**
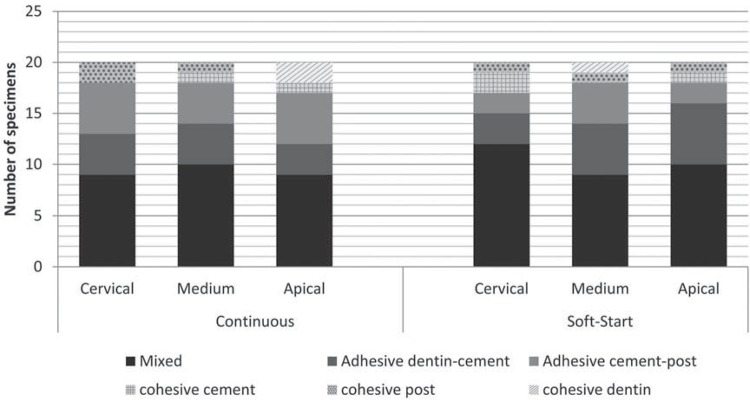
Absolute and Relative distribution (%) of the failure mode, considering the activation mode and the root regions

**Figure 2:**
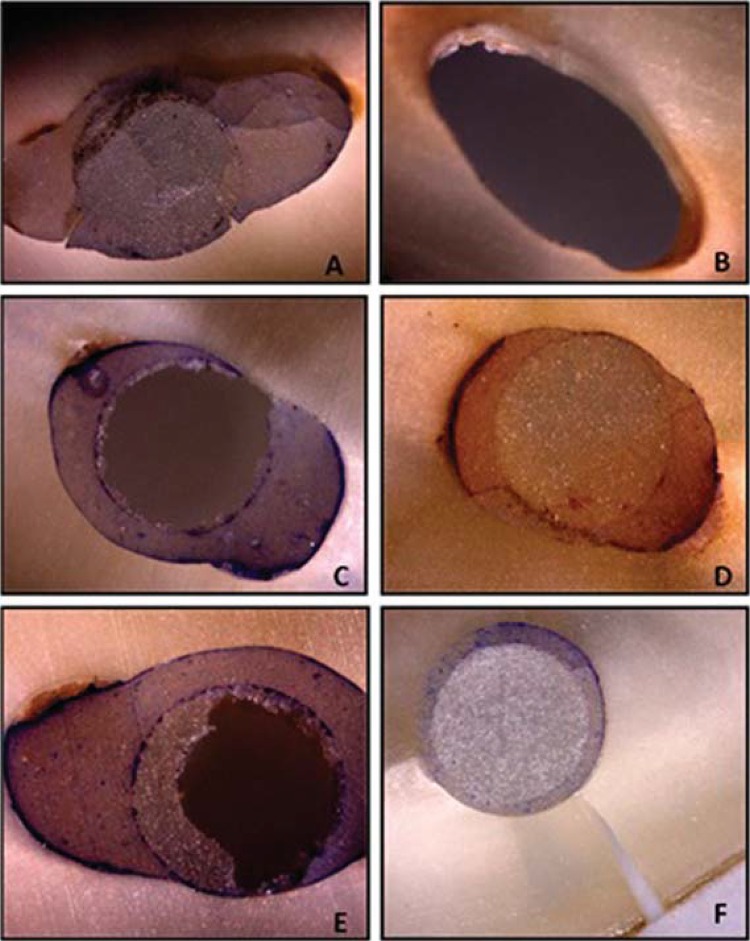
Optical Microscopy Representative (40X magnification) of each failure mode: A- mixed-failure; B- adhesive failure between cement-dentin; C- adhesive failure between cement-post; D- cohesive failure of cement; E- cohesive failure of post; F- cohesive failure of dentin

The absolute and relative distributions (%) of the failure modes are shown in Figure 1. Irrespective of the activation mode and root region, the mixed failure mode was the most prevalent. Representative images from the optical microscopy of each mode are illustrated in Figure 2.

## Discussion

Despite recent studies that have evaluated the shrinkage stress and activation protocol associated with fiber post cementation^6,23^, the literature is still limited regarding the use of different curing techniques and their effects on the aforementioned properties. In the present investigation we opted to use the push out bond strength test to evaluate the strength of the bonding between the fiber posts to the root canal under varying curing techniques, as this test closely simulates the clinical condition^15^. According to previous studies, the push-out test provides a better estimation of the bonding strength than the conventional shear test because the fracture occurs parallel to the dentin-bonding interface, which makes it a true shear test^15^.

Higher bond strength values were observed in the apical area of root canals irrespectively of the other factors. Although this is not in agreement with many studies published in this field^7^, it is in agreement with other authors^3,22^. The likely reason that explains such controversy is the type of resin cement employed in the different experimental designs. Studies that reported higher bond strength in the apical third^3^ employed self-adhesive resin cements while the others employed conventional, dual-cure luting materials^7^. The self-adhesive resin cement used (RelyX U200) is a new product, released as a substitute for the RelyX U100 resin cement. This resin cement has the same bonding mechanism of its predecessor, RelyX U100. Both products have micromechanical retention, but it seems that their bonding relies mainly on the chemical adhesion to hydroxyapatite^25^. This may be the rationale behind the product’s good performance in the apical third of the root canal in the present and earlier study^3^, different from what occurs with conventional resin cements.

It is known that the number of tubules greatly diminishes toward the apical region of the intraradicular dentin^11^. In the apical third of the root, there are fewer dentinal tubules^11^, the dentin is irregular, and it may be devoid of dentin tubules^9^. When present, these tubules are often sclerotic and filled with minerals that resemble those from peritubular dentin^26^. Altogether these factors increase the availability of calcium for chemical adhesion with the self-adhesive RelyX U200 in the apical region, which yields a higher bond strength at this third, as observed in this study. Conventional resin cements, on the other hand, rely mostly on micromechanical retention. Therefore, better bonding is expected to occur in areas with a high density of dentinal tubules, such as the cervical region^9^.

Another factor that favors self-adhesive cements, as claimed by manufacturers, is that that this type of resin cement was shown to be more tolerant to variations in dentin moisture, which is difficult to control in such areas of the root canal. While slight variations in the moisture degree of root dentin may jeopardize dentin bonding with conventional resin cement^13^, this had not yet been demonstrated for self-adhesive resin cements. Additionally, self-adhesive resin cements have both the characteristics of resin cement and glass ionomer cement. They have a rapid polymerization reaction initiated by light irradiation, and a slow acid-base reaction between the reactive glass filler and the acid functional monomer through water^20^. These materials suffer from effects of water sorption and solubility, which can influence strength, biocompatibility and the dimensional and color stability of polymeric-based cements^2^. A slight water sorption may have an essential effect in compensating the polymerization shrinkage of the resin, thus relieving internal stresses created during shrinkage and possibly improving marginal seal by decreasing gaps^12^.

During the cementation of endodontic posts to root canal dentin (in the worst case scenario) the C-factor exceeds 200^28^. Therefore, shrinkage stress in the confinement of the intact root canal may exceed the cement dentin bond strength, causing debonding of the cement from the dentin. This is a clear limitation of bonding fiber posts to root canals. The use of soft-start polymerization has been claimed to reduce the shrinkage stress by increasing the period that the resinous material remained in a low modulus of elasticity (pre-gel phase). This is possible through the low light intensity in the first seconds of light curing, which enables an accommodation of molecules and shrinkage stress relief by reducing the speed at which the polymerization occurs^17^.

This probably explains the higher bond strength values observed within the groups where soft-start polymerization was employed. Although a similar degree of conversion was observed in both activation methods, in the soft-start group debonding might have occurred less often than in the continuous mode. In the continuous activation mode, the light intensity remained constant from the beginning to the end of the polymerization process, decreasing in the pre-gel phase. This in turn produced a polymeric material with reduced flow capacity and potential for stress relief. In this circumstance, the low link became the adhesive interface, which may have debonded in some areas, producing low bond strength for this group^8,23^.

Another technique used in this study was micro-Raman spectroscopy, which has been proven to be well suited for the characterization of the chemical structure and characterization of adhesive resins, collagen and minerals at a resolution of up to 1 mm. It is also very useful in determining the degree of conversion of dental adhesives by providing a direct measurement of the percentage of converted double bonds^16,19^.

For light cured and dual cured resin cements, an adequate curing of the resin material by light is essential. Light intensity is higher at the cervical third^27^, yielding a higher degree of conversion than other regions^9,24^. This does not seem to be essential for self-adhesive resin cements, as the degree of conversion of Rely X U200 was neither affected by the curing technique, nor the root region. This was also observed in another published study^29^ that employed similar resin cement. These cited studies employed conventional, dual-cure resin cements and not self-adhesive cements, as were used in the present study.

Little has been published on the light-curing potential of conventional dual-cure cements. Earlier research suggests that auto-cure alone is not sufficient to achieve maximum cement hardening^7^ and this was also seen to be true for more recent resin-luting cements^21^. Perhaps, the dual-cure self-adhesive resin cements are capable of reaching maximum mechanical properties under light or chemical cure modes, explaining the similar degree of conversion observed in the different root regions. Additionally, both groups employed very similar energy densities, which may also be the reason for the similar degree of conversion between the two groups. However, further studies should be conducted in this field.

Regarding failure modes, there was no statistically significant difference between the two activation modes and root regions. The most frequent failure mode was the mixed type, which agrees with the results of some authors^1,13^ who used self-adhesive resin cement. The present study evaluated only one type of resin cement (dual self-adhesive), which does not necessarily reflect the general behavior of these groups. Thus, future studies should investigate more resin cements in order to investigate the differences between the classifications.

The present study has some limitations, for instance, no thermal cycling or mechanical stress was applied. These factors may limit the direct application of the study results to clinical conditions. Another limitation is that only one resin cement was employed to investigate the research question. As resin cements differ in their chemical and mechanical properties, caution should be used when applying the results of the present study to other materials available on the market.

## Conclusion

The bond strength of fiber posts to root canals can be improved by soft-started polymerization and the degree conversion was not affected by the curing mode of the resin cement.
